# Maize Stem Response to Long-Term Attack by *Sesamia nonagrioides*

**DOI:** 10.3389/fpls.2018.00522

**Published:** 2018-04-23

**Authors:** Victor M. Rodriguez, Guillermo Padilla, Rosa A. Malvar, Mario Kallenbach, Rogelio Santiago, Ana Butrón

**Affiliations:** ^1^Misión Biológica de Galicia, Spanish Council for Scientific Research (MBG-CSIC), Pontevedra, Spain; ^2^Centro de Investigaciones Biológicas, Spanish Council for Scientific Research (CIB-CSIC), Madrid, Spain; ^3^Department of Molecular Ecology, Max Planck Institute for Chemical Ecology, Jena, Germany; ^4^Departamento Biología Vegetal y Ciencias del Suelo, Facultad de Biología, Unidad Asociada BVE1-UVIGO, Universidade de Vigo, Vigo, Spain

**Keywords:** maize, omics, corn borer, plant defense, integrative analysis

## Abstract

Plants defend themselves against herbivores by activating a plethora of genetic and biochemical mechanisms aimed at reducing plant damage and insect survival. The short-term plant response to insect attack is well understood, but less is known about the maintenance of this response over time. We performed transcriptomic and metabolomics analyses in order to identify genes and metabolites involved in the long-term response of maize to attack by the corn borer *Sesamina nonagrioides*. To determine the role of elicitors present in caterpillar secretions, we also evaluated the response of maize stem challenged with insect regurgitates. The integrative analysis of the omics results revealed that the long-term response in maize is characterized by repression of the primary metabolism and a strong redox response, mainly mediated by germin-like proteins to produce anti-nutritive and toxic compounds that reduce insect viability, and with the glutathione–ascorbate cycle being crucial to minimize the adverse effects of reactive oxygen species (ROS) on the plant. Our results suggest that different defense mechanisms are involved in the long-term response compared to those reported during the early response. We also observed a marginal effect of the caterpillar regurgitates on the long-term defensive response.

## Introduction

Plants and phytophagous insects are constantly interacting in natural and agricultural environments. Upon recognition of predators, plants activate inducible defenses in order to avoid extensive damage that could compromise their survival. Due to resource restrictions, plants have to constantly balance the allocation of resources to growth and defense and thus inducible defenses must be precisely regulated. This fine-tuning is reflected in specific plant responses to different insect challenges (Bonaventure et al., 2011). Most studies have focused on the elucidation of the mechanisms underlying plant responses to folivore insects; in contrast, little is known about plant-defense mechanisms induced by herbivores that use different feeding strategies or feed on different parts of the plant.

The term “stalk or stem borer” encompasses a number of Lepidopteran caterpillars belonging to three insect families (Noctuidae, Crambidae, and Pyralidae), among which *Sesamia nonagrioides* (Lef) is the prevalent maize pest in the Mediterranean region (Cordero et al., 1998). The most common life cycle of these arthropods shows from two to four generations per year. Larvae of the first generation are scarce and feed on the whorl of juvenile plants, causing plant death or delayed development. Subsequent generations are more numerous and feed on adult plants. Larvae penetrate into the stalk and feed in the stem pith, resulting in crop losses, early leaf senescence, and interference with translocation of metabolites. Larval feeding on stems elicits an early response, largely mediated by phytohormones; stem tissue undergoes extensive transcriptome reprogramming that triggers the expression of protease inhibitors, secondary signal transduction components, pathogenesis-related genes, and transcription factors involved in defensive responses to slow down herbivore development and to limit tissue damage (Zhou et al., 2011; Rodríguez et al., 2012; Dafoe et al., 2013).

Transcriptomic reorganization is eventually reflected in metabolomic changes and substantial modifications of plant cell physical structures. Several metabolites have been associated with maize resistance to attack by stem borers. Short-term herbivory by corn borers has been shown to enhance the accumulation of some antibiotic compounds (e.g., benzoxazinoids and kauralexins). Nevertheless, tissue susceptibility to insect feeding was increased because such changes were not sufficient to counteract the increased availability of soluble protein, sucrose, and linolenic acid from the challenged stem tissue (Malvar et al., 2008; Dafoe et al., 2011, 2013; Schmelz et al., 2011). In parallel, the cell wall has been identified as the main physical barrier to maize attack by stem borers (Malvar et al., 2008). The cell wall acts as a passive physical barrier to restrict insect penetration and renders nutrients within tissues less accessible (Santiago et al., 2008). However, new transcriptome and biochemical evidence has revealed that the maize stem responds to attack by stalk borers by activating genes and inducing the synthesis of compounds involved in cell-wall strengthening (e.g., diferulic acid and lignin) (Rodríguez et al., 2012).

The early plant response to insect attack has been comprehensively studied in many plant–insect interactions. Early maize responses to leaf feeding by chewing and sucking insects have been the subject of many investigations using transcriptomic, proteomic, and metabolomic approaches (Marti et al., 2013; Tzin et al., 2015; Guo et al., 2017). However, fewer studies have focused on the late maize response to stem attack by corn borers, although it has been demonstrated that the long-term plant response to insect chewing could be stronger than the early response and become systemic (Stout et al., 2009). Many authors have also observed a clear difference in plant response to sucking insects following short- versus long-term exposure (Gutsche et al., 2009; Tzin et al., 2015; Uawisetwathana et al., 2015; Donze-Reiner et al., 2017). In particular, the antibiotic effect triggered by corn borers only seems to be relevant in the long-term response, and changes in the response to short- versus long-term attack, due to continuous cross-talk between the plant and the larvae, could contribute to increased antibiosis over time (Santiago et al., 2017).

Plants could perceive the presence of herbivores through touch, e.g., insect crawling on plant surface or egg deposition, but it is generally accepted that a combination of both, mechanical damage and the delivery of chemical elicitors abundant in the insect saliva, is responsible for the full plant response to insect herbivores (Reymond et al., 2000; Hall et al., 2004; Engelberth et al., 2007; Little et al., 2007). Interestingly, most of the compounds present in insect secretions are not elicitors of general responses in all plant species but are usually restricted to particular plant–insect associations (Bonaventure et al., 2011). Lepidopteran caterpillars produce two types of secretions: oral secretions (OSs) produced in the digestive system and saliva produced in the salivary glands (Felton, 2008). The previously untested secretions of *S. nonagrioides* were tested in the current study to gain more insight into the induction of stem defenses by stem borer secretions and to complement the few available studies reporting activation of maize defenses after elicitation with stem borer secretions (Dafoe et al., 2013; Louis et al., 2013).

Understanding the complexity of the plant defensive response requires the analysis of all metabolic pathways within a plant with potential activity against herbivores, rather than focusing on a particular subset. In this context, the development of new omics tools has made it possible to obtain large amounts of metabolomic, proteomic, and transcriptomic data involved in plant response to insect attack. The integration of data obtained from these multidisciplinary omic approaches is the current challenge. We used a multi-omic approach to elucidate the long-term response of maize plants to attack by the corn borer *S. nonagrioides*. Maize plants were also treated with regurgitate obtained from *S. nonagrioides* larvae to differentiate the plant response due to effectors and elicitors present in the insect regurgitate from that due to mechanical chewing.

## Materials and Methods

### Plant Material and Experimental Design

Seeds of the inbred line PB130 were sown in pots and grown under greenhouse conditions. In a previous report, we evaluated the early transcriptomic response of this resistant inbred line to attack by *S. nonagrioides* (Rodríguez et al., 2012). At maize silking, each treatment [infestation with *S. nonagrioides* larvae, wounding, wounding + regurgitate, and control (untreated plants)] was applied to three different plants (biological replicates). The plants were infested by laying three 2nd instar larvae of *S. nonagrioides* on each maize plant. Larvae were placed between the leaf sheath and the stem, in the first and second internodes below the main ear. Wounding was performed with a scalpel; three wounds (scratches) of 5 × 5 mm were made to the leaf sheaths below the main ear. After wounding, 5 μl of 0.1 M phosphate buffer solution (PBS) or *S. nonagrioides* regurgitate suspended in PBS was applied using a micropipette to each wound, depending on the treatment. The wound site was sealed with parafilm to reduce drying. Wounding and wounding + regurgitate application were repeated every other day. To obtain insect regurgitates, 50 *S. nonagrioides* larvae were gently pinched with tweezers, causing the regurgitation of approximately 2 μl that was re-suspended in phosphate buffer and kept frozen at -80°C until use.

Fifteen days after treatment application, a section (free of damage) of approximately 2 cm in the upper part of the stalk internode just below the ear was collected from each plant. The leaf sheath was discarded. Samples were immediately frozen in liquid nitrogen and conserved at -80°C.

### Fatty Acid/Amino Acid Conjugate Analysis of the Regurgitate

Fatty acid/amino acid conjugates (FACs) were analyzed from 10 μl of *S. nonagrioides* regurgitate, diluted 10-fold with 70% MeOH (v/v). Nonadecylyl-L-glutamine (C19:0-Gln) was used as the internal standard [500 pg/μl (m/v)]. A 10 μl sample was injected into an LC–ESI–MS/MS instrument (Varian 1200 Triple-Quadrupole–LC–MS system; Varian Inc., Walnut Creek, CA, United States). Mobile phases were (A) H_2_O containing 0.05% (v/v) formic acid (FA) and (B) methanol. The following gradient was used: 0–2.5 min 15% B (v/v), 2.5–4.5 min 15–98% B, 4.5–17.5 min 98% B, 17.5–18.5 min 98–15% B, and 18.5–20 min 15% B. A 50 × 2 mm, 120 Å, C18 column (Phenomenex, Aschaffenburg, Germany) attached to a precolumn (C18, 4 × 2 mm, Phenomenex, Aschaffenburg, Germany) was used with a flow of: 0–1.0 min 0.4 ml min^-1^, 1.0–1.5 min linear gradient from 0.4 to 0.2 ml min^-1^, 1.5 to 15 min 0.2 ml min^-1^, 15 to 15.5 min 0.4 to 0.2 ml min^-1^, and 15.5 to 20 min 0.4 ml min^-1^. To minimize contamination, only the solvent eluting from the column between 1.5 and 15.5 min was injected into the mass spectrometer. Between 0 and 1.5 min and 15.5 and 25 min, a mixture of 1/1 (v/v) MeOH/H_2_O was injected to flush the MS/MS system. Detection was performed in negative ionization mode using multiple reaction monitoring (MRM) after collision-induced dissociation (CID) with argon gas. For ionization, the needle was set to 5000 V and the drying gas (N_2_) to 300°C and 20 psi (housing 50°C). The detector was set to 1400 V. Quantification was based on the peak area of the internal standard C19:0-Gln. The MS Workstation software version 6.8 (Varian Inc., Walnut Creek, CA, United States) was used to control the instrument and calculate peak areas.

### Metabolomic Profile

Metabolic profile was obtained in three biological replicates per treatment. Metabolites of each maize sample were isolated from 100 mg of frozen ground tissue in 1 ml of extraction buffer [50 mM of acetic acid in 40% MeOH (v/v)]. Samples were vortexed for 20 min and centrifuged at 20,000 × g/4°C. Extracts were filtered through a 0.22-μm membrane to remove particles. A 5 μl sample was injected into a UPLC–QTOF instrument (Waters HDMS Synapt; Waters Inc., Milford, MA, United States). Mobile phases were (A) H_2_O containing 5/0.1% (v/v) acetonitrile/FA and (B) acetonitrile containing 0.1% FA. The following gradient was used: 0–17.3 min, linear gradient from 0 to 22% B (v/v), 17.3–27.3 min 22–28% B, 27.3–28 min 28–40% B, 28–28.5 min 40–100% B, 28.5–30 min 100% B, 30–30.5 min 100–0% B, and 30.5–32 min 0% B, using a 100 × 2.1 mm, 1.7 μm UPLC ethylene-bridged hybrid (BEH) C18 column (Waters Acquity, Waters Inc., Milford, MA, United States) with a flow of 0.3 ml min^-1^. The following settings were applied during the LC–MS runs: capillary voltage at 3.0 kV, cone voltage at 28 eV, and collision energy at 4 eV. For the LC–MS/MS runs, collision energy was set to 10–25 eV. Both negative and positive ionization modes were selected using the electrospray ionization (ESI) interface. The MassLynx Software version 4.1 (Waters Inc., Milford, MA, United States) was used to control the instrument and calculate accurate masses. Ion abundance with a *P*-value ≤ 0.05 (one-way ANOVA) and a -1 ≤ logFC ≥ 1 were considered differentially accumulated in both conditions. The metabolomics data were submitted to the MetaboLights repository (Accession No. MTBLS575).

### RNA Isolation, Library Preparation, and Sequencing

Individual sample tissues (three biological replicates per treatment) were ground in liquid nitrogen and the RNA was extracted using the Spectrum^TM^ Plant Total RNA Kit (Sigma–Aldrich, St. Louis, MO, United States). To remove any traces of genomic DNA, the RNA was treated with DNase. Twelve massive analysis of 3′-cDNA ends (MACE) libraries, one from each sample, was constructed by GenXPro GmbH (Frankfurt am Main, Germany). Transcript libraries were constructed as described by Zawada et al. (2014). Briefly, poly-adenylated mRNA was isolated from 1 μg of total RNA and cDNA was produced by first and second strand synthesis using the SuperScript^®^ III First-Strand Synthesis System (Life Technologies GmbH) with modified barcoded poly-T adapters that are biotinylated at the 5′-end. After cDNA random-fragmentation, the 3′-ends were captured by streptavidin beads and the 5′-ends of ≈250-bp-long fragments from the 12 barcode samples were sequenced (single-read) using an Illumina Hiseq 2000 version 4 (Illumina, Inc., San Diego, CA, United States), with 1 × 125 bp (6 bp are used for a barcode). In order to avoid PCR-bias differences in amplification efficiency, GenXPro’s “TrueQuant” method was applied. Briefly, this method identifies and eliminates copies with an identical barcode-sequence combination from the generated dataset (Yakovlev et al., 2014).

Low-quality ends and adapters were removed from the reads using Cutadapt (Martin, 2011). Cleaned and trimmed reads were processed according to the following sequential pipeline:

(1) Assembly and annotation to the ENSEMBL maize genome, release 23^1^.
(2) Annotation to the transcriptome from the same release with those reads for which no information could be retrieved after (1).
(3) Assembly of all reads with no annotation after (1) and (2) and BLASTX with the generated contigs to the swissprot database.
(4) BLASTN with the generated contigs after (3) to all plant transcriptomes.

Assemblies were performed with NovoAlign v3.00.05^2^; reads mapping to multiple locations were limited to 10. All other options were set at default. Normalization and differential expression analysis were performed using the R package DESeq (Anders and Huber, 2010) with the default parameters. Gene Ontology (GO) annotations and enrichment analyses were performed independently for the up- and down-regulated genes within each comparison with the online webserver Plant GeneSet Enrichment Analysis toolkit (Yi et al., 2013). Besides GO gene sets, this analysis includes two other categories: Gene family-based gene sets (GFam) from the Plant Transcription Factor and the ubiquitin proteasome system (PlnTFDB/plantsUPS) databases and curated gene sets from the PlantCyc. The KEGG ortholog annotation (KO) and identification of significant pathways were carried out using the KOBAS 2.0 server^3^ (Xie et al., 2011), with the UniProtKB accession numbers being used as the input terms. Therefore, differentially expressed model genes with no loci linked to them were not included in the analyses. Gene enrichment analyses in particular pathways were performed for each comparison, but including up- and down-regulated genes.

Transcripts/genes with a false discovery rate (FDR) < 0.05 were considered differentially expressed between conditions. The whole transcriptomic dataset was submitted to the Gene Expression Omnibus (GEO) repository (Accession No. GSE107133).

### Data Integration: O2PLS Model

In order to integrate the transcriptomic and metabolomics data, we performed an O2PLS analysis. This method decomposes the variation present in the two data matrices into three parts, the joint variation between the two datasets, the orthogonal variation that is unique to each dataset and noise. The model assumes that some latent variables are responsible for the variation in the joint and orthogonal parts (Bouhaddani et al., 2016). Pre-processing of the datasets was performed as described in Bylesjö et al. (2007). Briefly, both datasets were mean-centered per feature element and the metabolomics data were scaled to unit variance for each resolved peak. Finally, both datasets were scaled to an equal total sum of squares of 1. For better interpretation of the data, only features that were considered significant in individual transcriptomic and metabolomics analyses were considered during the integration analysis.

O2PLS models were calculated using the OmicsPLS package of R (Bouhaddani et al., 2016). To establish the thresholds for identifying the most influential variables, we performed a permutation test. Datasets were reshuffled 1000 times and the O2PLS model was generated for each rearranged dataset. Considering a significant level (α) of 0.05 for both datasets, the upper and lower α/2 quantiles of the loading values were used as thresholds for the latent variables.

## Results

### Identification of Differentially Expressed Genes in Response to Insect Feeding

**FIGURE 1:**
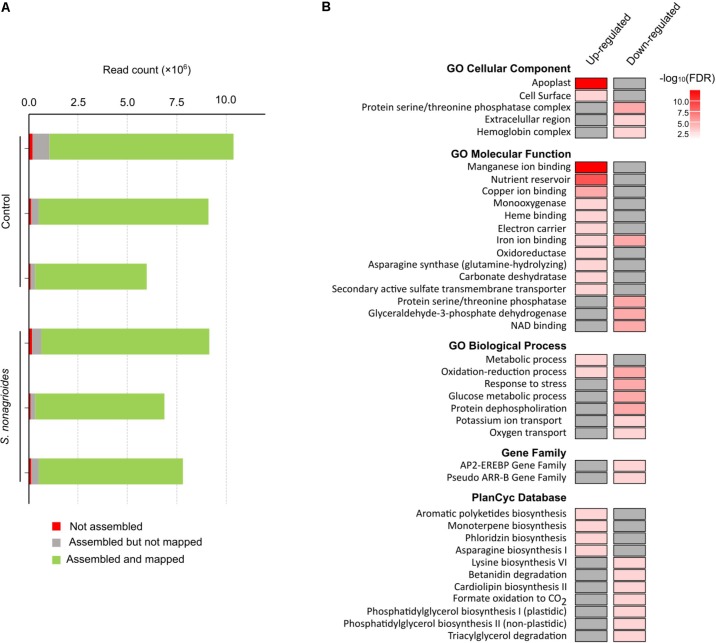
Transcriptomic response to long-term maize challenge with *S. nonagrioides* caterpillars based on results from three biological replicates per treatment. **(A)** Number of reads identified from the six libraries sequenced. **(B)** Enriched GO terms, family-based gene sets (gene family), and PlantCyc curated database terms (FDR < 0.05).

After removal of low-quality reads, between ∼6 and ∼10.5 million 119-nt reads were detected in each library (**Figure 1A**). Around 95–99% of the reads were assembled and the percentage of these assembled reads that mapped to the maize reference genome ranged from ∼92% to ∼97%. The assembled and mapped tags gave 50,939 transcripts, but only 29,726 of them corresponded to annotated genomic regions.

A total of 126 genes were differentially expressed: 54 were up-regulated and 72 were down-regulated (Supplementary Table 1). The major group of genes differentially expressed after *S. nonagrioides* feeding comprised genes involved in reactive oxygen species (ROS) production and scavenging (peroxidases, laccases, and *cupin-like* genes). Most of these genes were up-regulated, although a significant number of them were down-regulated after treatment. On the contrary, most of the genes involved in plant defense against biotic stresses, including some transcription factors, were down-regulated. Just a few pathogen-related (PR) and SAR-signaling-related genes were up-regulated. A gene family-based annotation analysis (GFam) indicated that genes that code for ethylene-responsive proteins with an APETALA2 DNA-binding domain and pseudo *ARR-B* genes were over-represented in the group of genes repressed by *S. nonagrioides* attack (**Figure 1B**). Genes involved in primary metabolism [i.e., carbohydrate transport (nodulin-like genes), metabolism of carbohydrates, and lipids] were down-regulated by insect feeding.

Gene Ontology analysis revealed significant up-regulation of genes encoding for proteins located on the cell surface and especially in the apoplast, while genes located in the protein serine/threonine phosphatase complex, the extracellular region, and the hemoglobin complex were down-regulated (**Figure 1B**). According to molecular function and biological process categories, genes in the nutrient reservoir activity and manganese ion-binding GO categories were up-regulated, whereas genes involved in glucose metabolism and de-phosphorylation processes were down-regulated (**Figure 1B**).

### Metabolic Profile of the Maize Stem in Response to Insect Feeding

**FIGURE 2:**
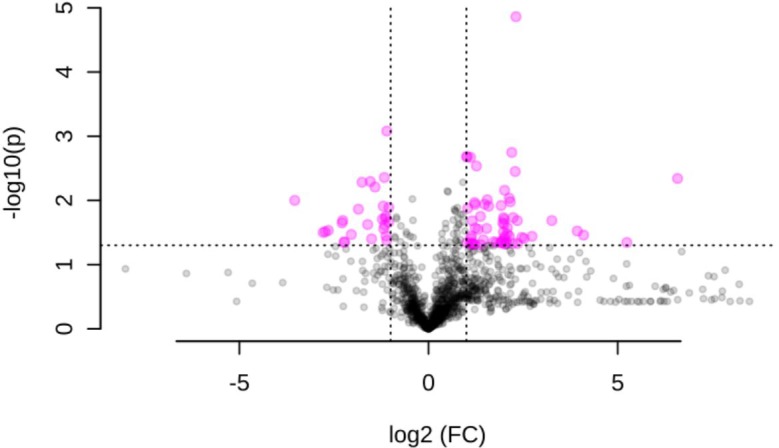
Volcano plot of the metabolomics response to long-term maize challenge with *S. nonagrioides* caterpillars based on results from three biological replicates per treatment. Significant metabolites (*P*-value < 0.05 and a –1 < log_2_FC > 1) are represented in pink color.

When plants were challenged with *S. nonagrioides* caterpillars, 85 ions were differentially accumulated (**Figure 2** and Supplementary Table 2). Fifty-three were identified in positive, 26 in negative, and 5 in both ionization modes. A list of metabolites previously identified as being involved in defense against attack by Lepidopteran larvae (Supplementary Table 3), along with publically available databases, was used to annotate the 85 differentially accumulated ions. Twenty-nine molecular formulas could be assigned to the set of differentially accumulated ions. Approximately 50% of these molecular formulas could be significantly associated with a maximum of two metabolites in the databases.

The concentration of several antioxidant molecules, including low mass molecular thiols (i.e., glutathione and L-Cys–Gly; NADH, which acts as a cofactor of several antioxidant enzymes; and the phenylpropanoid Safflomin C), decreased after *S. nonagrioides* feeding. Another putative compound that decreased after insect feeding, 2,4-dihidroxi-7-metoxi-1,4-benzoxazin-3-ona (DIMBOA), is a benzoxazinoid with known insecticidal properties. A significant accumulation of DIMBOA-glucoside was observed although the logFC did not exceed 1. However, increases in metabolites potentially involved in plant defense were also observed. Some of these metabolites could be classified as alkaloids based on their molecular formula and their possible structures. Four ions that increased after insect attack could be assigned to compounds other than alkaloids that are likely involved in plant defense. Two of these compounds have been associated with plant defense against fungi: indoleacrylic acid (an auxin derivate) and 4-*O*-β-glucosylsinapate (a phenylpropanoid compound, and in particular, a monolignol glucoside) (Matchett, 1972; Yogendra et al., 2014). Another ion could be associated with Sesartemin, which is a lignan that has been demonstrated to inhibit monooxygenase activity in the gut of the European corn borer (*Ostrinia nubilalis*) (Bernard et al., 1989). The last ion could be associated with two compounds, *trans*-aconitate and dehydroascorbic acid. *Trans*-aconitate is synthesized in response to a wide range of stresses either biotic or abiotic, and plants of the Poaceae family are especially rich in this compound (Katsuhara et al., 1993; Rémus-Borel et al., 2009), whereas dehydroascorbic acid acts in plant protection against oxygen-free radicals (Rautenkranz et al., 1994).

### Multiomics Integrative Analysis of the Response to Insect Feeding

**FIGURE 3:**
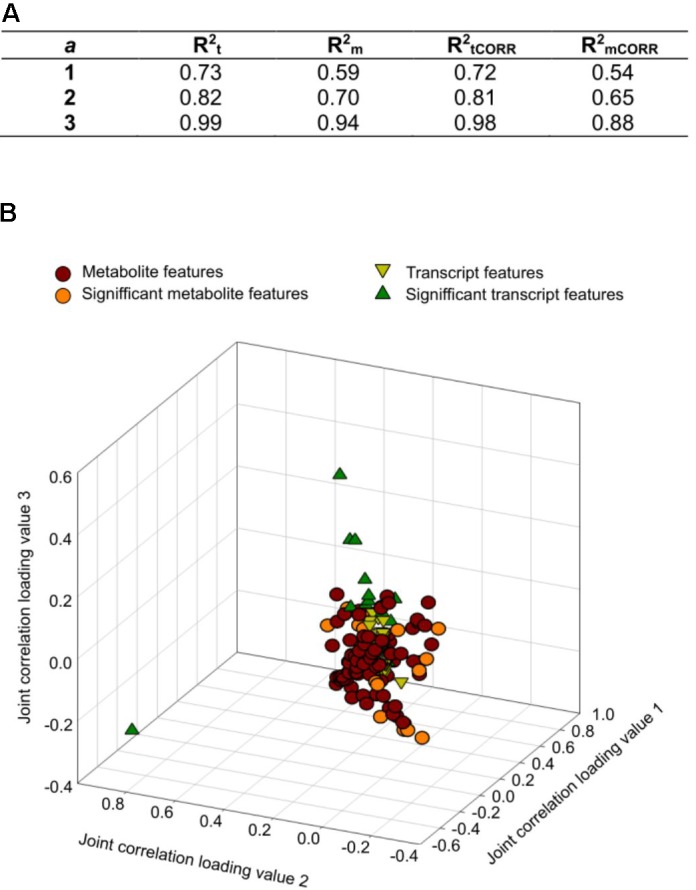
Integrative O2PLS analysis of significant features identified from the transcriptomic and metabolomics analysis of the maize stem after *S. nonagrioides* feeding. **(A)** *R*^2^ for each statistic. The number of orthogonal components was held to 1. The number of joint components varies from 1 to 3. **(B)** Loading values representation of transcripts and metabolites for the three first joint components.

Integration analysis was performed using from one to three joint components and one orthogonal component per dataset (transcriptomic and metabolomics). The model used with three joint components explained more than 90% of the total variation of the transcriptome ($R_{t}^{2}$) and metabolome ($R_{m}^{2}$) (**Figure 3A**). Almost 100% of the modeled transcriptome and metabolome variations were considered joint variation because they could be explained by variation in the complementary model [metabolomics ($R_{mCORR}/R_{m}^{2}$) and transcriptomic ($R_{tCORR}/R_{t}^{2}$) models, respectively]. Based on the loading coefficients thresholds calculated after 1000 permutations, we identified 10 transcripts and 16 metabolites as having the most influence on the model (**Figure 3B**). The induced transcripts with the highest influence on the metabolome code for proteins involved in response to stresses and cell detoxification, whereas those that were repressed are associated with carbohydrate metabolism and transport, synthesis of cell wall proteins, and biosynthesis of ethylene (**Table 1**). Unfortunately, ion identification remains a bottleneck in metabolomics analysis and few features could be assigned to specific compounds. This makes it difficult to obtain a general picture of the joint variation. Three relevant ions could be assigned to specific formulae; one ion was putatively assigned to a phytohormone, another to a detoxification compound, and the third one to two alternative substances, one with antioxidant properties and the other being an intermediate in the synthesis of purines (**Table 2**).

**Table 1:** Transcripts with significant loading coefficient in the integrative O2PLS analysis after *S. nonagrioides* feeding.

Gene ID	Expression	Description	Biological function
GRMZM2G042789	Induced	Subitilisin-chymotrypsin inhibitor	Defense response
GRMZM2G044383	Induced	Glutathione-*S*-transferase	Detoxification
GRMZM2G320373	Induced	Nonspecific lipid-transfer protein AKCS9	Lipid transfer
GRMZM2G028104	Repressed	10-Deacetylbaccatin III acetyl transferase	Terpenoid-derived alkaloid biosynthesis
GRMZM2G176307	Repressed	Glyceraldehide-3-phosphate dehydrogenase 4	Carbohydrate catabolism
GRMZM2G172204	Repressed	β-Glucosidase aggregating factor 1 (bgaf1)	Carbohydrate catabolism
GRMZM2G071630	Repressed	Glyceraldehide-3-phosphate dehydrogenase 3	Carbohydrate catabolism
GRMZM2G147399	Repressed	Early nodulin 93	Carbohydrate transport
GRMZM2G014705	Repressed	Orthologous to a putative early nodulin 93 gene in rice	Carbohydrate transport
GRMZM2G067315	Repressed	Glycine-rich cell wall structural protein 2	Cell wall protein
GRMZM2G168651	Repressed	Hydroxyproline-rich glycoprotein 1	Cell wall protein
GRMZM5G843555	Repressed	Procollagen-proline dioxygenase	Cell wall protein
GRMZM2G013448	Repressed	Aminocyclopropane-carboxylate oxidase	Hormone biosynthesis and perception


**Table 2:** List of identified metabolites with significant loading coefficient in the integrative O2PLS analysis after *S. nonagrioides* feeding.

Exact mass	Expression	Putative formula	Description
337.056	Repressed	C15H14O9/C9H15N4O8P	2-O-Acetyl-*trans*-coutaric acid/5-aminoimidrazole-4-carboxamide-1-β-D-ribofuranosyl 5′-monophosphate
306.076	Repressed	C10H17N3O6S	Glutathione
339.074	Repressed	C15H15CIN2O5	*N*-(Carbomethoxyacetyl)-4-*S*-chlorotryptophan (Chloroindole auxin)


### Qualitative Analysis of FACs in the Insect Regurgitates

In order to determine the presence of elicitors in *S. nonagrioides* regurgitate, we performed a LC–MS/MS analysis. We identified 12 FACs in the *S. nonagrioides* regurgitate (**Table 3**). Similar to other noctuids, *N*-(17-hydroxylinoleoy)-L-glutamine (named Volicitin) was present in the caterpillar regurgitate. Most of the identified FACs derived from oleic (18:1), linoleic (C18:2), and linolenic (18:3) acids. It is worth mentioning that only derivates of linolenic acid are considered active as plant elicitors on maize seedlings (Alborn et al., 1997). The amino acid moiety is also important in order to induce the production of plant volatiles (Mori et al., 2003). Two types of amino acid have been reported to be constituents of FACs: glutamic acid and glutamine. Only traces of FACs with glutamic acid moiety were present in *S. nonagrioides*. These FACs are considered the oldest in evolutionary terms (Mori et al., 2003).

**Table 3:** Concentration of Fatty acid-amino conjugates (FACs) identified in the S. nonagrioides regurgitate.

Compound	RT (min)	MS^1^ (*m*/*z*)	MS^3^ (*m*/*z*)
OH-C18:3-Glu	1.8	381	145.0
OH-C16:0-Glu	2.4	425	145.0
OH-C18:2-Gln	10.9	426	297.0
OH-C18:1-Glu	11.4	423	145.0
OH-C18:2-Glu	11.1	421	145.0
C16:0-Gln	12.6	383	144.9
C18:1-Gln	12.8	409	145.0
OH-C18:1-Gln	11.2	400	271.0
C18:3-Gln	11.9	405	145.0
OH-C18:3-Gln (Volicitin)	10.8	424	295.0
C16:1-Gln-isomer1	12.1	423	145.0
C18:2-Gln	12.3	407	145.1


*RT, retention time.*

### Few Common Features Were Observed Between the Response to Insect Feeding and Insect Regurgitate Treatments

**FIGURE 4:**
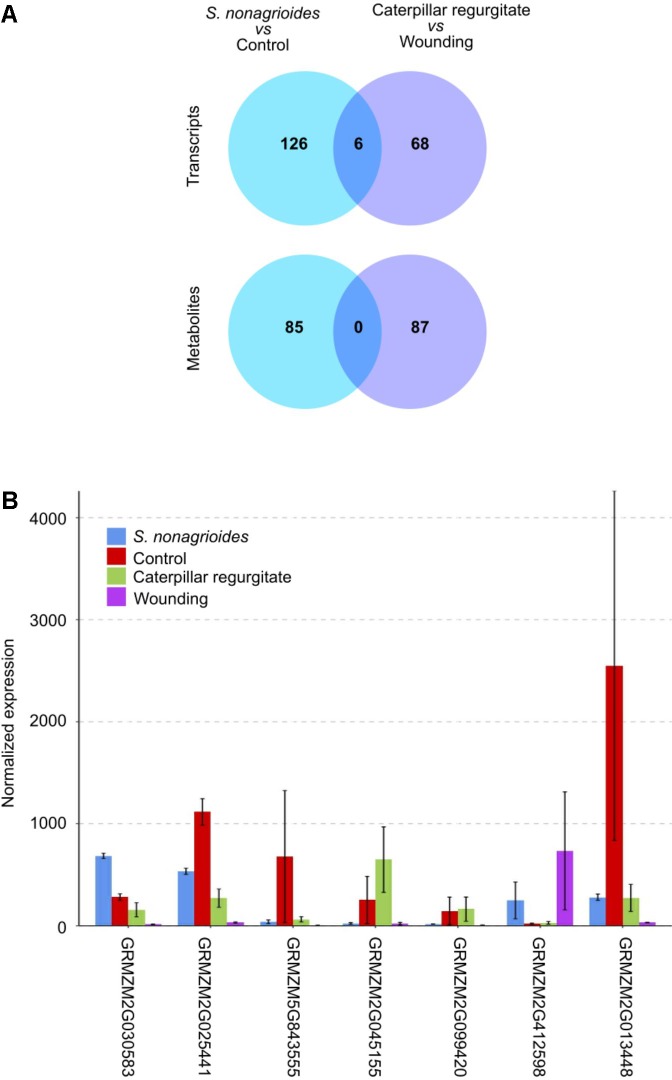
Comparison of the maize transcriptomic and metabolomics long-term response to *S. nonagrioides* feeding and challenge with caterpillar regurgitates. **(A)** Venn diagram of transcripts and metabolites identified at both treatments. **(B)** Expression of the seven genes commonly identified at both treatments. Error bars represent ±*SD*.

The comparative analysis of the maize response to insect feeding and to caterpillar regurgitates indicated that only seven genes and not a single metabolite were simultaneously implicated in both responses (**Figure 4A**). Curiously, only the transcript GRMZM2G030583, which codes for a *monoterpene synthase* (*tps26*), was over-expressed in both responses. The remaining common transcripts showed opposite behavior in response to the two stimuli. The expression of genes GRMZM2G025441, GRMZM5G843555, GRMZM2G013448, GRMZM2G045155, and GRMZM2G099420 was down-regulated after *S. nonagrioides* feeding but up-regulated after insect regurgitate challenge, whereas the opposite behavior was observed for the transcript GRMZM2G412598 (**Figure 4B**).

In general terms, the response to caterpillar regurgitate treatment was characterized by the repression of genes encoding for osmoprotective proteins and several genes of the chalcone synthase family, which could be involved in the synthesis of phenolic lipids. Genes involved in the phenylpropanoid pathway; protein, carbohydrate, and lipid metabolisms; terpenoid biosynthesis; synthesis and catabolism of phytohormones; and signal transduction systems (e.g., cell wall glycoproteins, protein kinases, and tubulin genes) were stimulated (Supplementary Table 4). The metabolomic response was poorly characterized because few metabolites could be putatively identified. Among those identified, increases in compounds with antioxidant properties were observed (Supplementary Table 5). Three of these compounds were classified as flavonoids (4′, 6′-dihydroxy-2′-methoxyacetophenone 6′-glucoside, epicatechin 5-O-β-D-glucopyranoside-3-benzoate, and cyanidin 3-*O*-3″,6″-*O*-dimalonylglucoside). The other two compounds were the riboflavine tetrabutanoate, which is a hydrophilic derivate of riboflavin that is widely distributed in nature, and ascorbyl palmitate, which is a derivate of ascorbic acid with antioxidant properties (Cort, 1974). Another two putative compounds identified were products of fungal metabolism, the mycotoxin T_2_ produced by *Fusarium sporotrichioides*, which is capable of infecting maize (Moya-Elizondo et al., 2013), and 9,10-dihydroergocornine methanesulfonate, an ergot alkaloid produced in the sclerotia of *Claviceps* species, which are common pathogens of various grass species (Bennett and Klich, 2003).

## Discussion

In a previous study, we demonstrated that the maize response to stem chewing by *S. nonagrioides* relies on complex transcriptomic networks that are genotype-dependent (Rodríguez et al., 2012). To analyze the long-term stem response to corn borer feeding, we used the inbred line PB130. This inbred line shows remarkable resistance and a strong inducible response to insect attack. In the previous study, several days elapsed from the initiation of insect feeding to maize sample collection whereas 2 weeks separated these events in the current study. We shifted the focus of the study toward the plant’s reaction to long-term attack because some defenses are not expressed until days to weeks after induction (Rasmann et al., 2012; Tian et al., 2012).

### Different Defense Mechanisms Are Involved in the Long-Term Response Compared to Those Reported on the Short Term

Consistent with other studies, our data indicated that different defense mechanisms could be involved in the response to long-term feeding by stem borers compared to those implicated in the early response (Gutsche et al., 2009; Uawisetwathana et al., 2015; Donze-Reiner et al., 2017). The early response to feeding by corn borers was characterized by the activation of signaling mechanisms mediated by phytohormones (Dafoe et al., 2011; Rodríguez et al., 2012), whereas our data suggested only marginal involvement of these molecules in the long-term response. We did not observe any differential expression of genes involved in JA biosynthesis, whereas several transcripts involved in ABA and ethylene biosynthesis and signaling were repressed after *S. nonagrioides* feeding, including three members of the AP2-EREBP transcription factor family. It seems that the balance between ABA and ET signaling fine-tunes the JA-mediated defenses induced by insect herbivores, with ET acting more as a modulator of the induced response than a direct elicitor (Nguyen et al., 2016). As ET could act synergistically with JA against some diseases, the initial contact with insect regurgitate, charged with a plethora of microbes, could increase ET biosynthesis, while an antagonistic relationship between JA and ET could be established later on to correctly fine-tune a more specific response against herbivores (Mantelin et al., 2009; Chung et al., 2013; Song et al., 2014).

Induction of phytohormones in the early response was accompanied by the accumulation in the stem tissue of compounds with antibiotic effect, essentially benzoxazinoids and kauralexins (Dafoe et al., 2011, 2013; Schmelz et al., 2011). On the contrary, the *de novo* synthesis of benzoxazinoids did not appear to be stimulated after long-term feeding. The tendency to increase DIMBOA-Glc did not compensate for the significant reduction in DIMBOA and no conversion toward derivate compounds with increased antibiotic effect such as HDMBOA-Glc was observed. These data suggest a reduced role of stem benzoxazinoids in the long-term maize defenses against corn borers, in contrast to results reported for the early response (Dafoe et al., 2011). Similarly, no involvement of induced kauralexins in maize stem defenses after long-term feeding by corn borers was observed, although three genes involved in the synthesis of terpenoid compounds were up-regulated by insect feeding. In contrast, we observed the accumulation of several probable alkaloid compounds after feeding by *S. nonagrioides* larvae, although no up-regulation of genes involved in alkaloid biosynthesis was observed. However, increased levels of alkaloids could be regulated by the availability of the amino acid substrates rather than by increased expression of biosynthesis genes (Zhang et al., 2009). In the current study, amino acid disposal may have been stimulated by insect attack because peptidase and asparagine synthetase transcripts were upregulated. We propose that asparagine could act as a nitrogen transporter and reservoir for systemic alkaloid biosynthesis.

### The Long-Term Defensive Response Is Characterized by the Repression of Plant Primary Metabolism and Activation of ROS Scavenging Machinery

In an effort to integrate the metabolomics and transcriptomic data, we performed an O2PLS analysis. This analysis indicated that two groups of genes had the highest impact on the metabolome. The first group included genes involved in primary metabolism and plant growth that were down-regulated after *S. nonagrioides* feeding. These results are consistent with the fact that maize growth is affected by borer feeding even before the larvae enter into the stalk: leaves become smaller, internodes shorter, and tasseling and silking are delayed (Chiang and Holdaway, 1959). The impact of the plant primary metabolism on insect *O. nubilalis* growth was reported by Dafoe et al. (2013). These authors observed an increase in the nutritional value of the maize stem after 48 h of feeding that rendered maize stems more susceptible to borer feeding. Based on this, we hypothesize that decreasing the primary metabolism, especially the metabolism of carbohydrates and lipids, would render the stem tissue less nutritious for insects. In our previous study, the primary metabolism did not seem to be affected by medium-term feeding by *S. nonagrioides* larvae (Rodríguez et al., 2012), suggesting that the reduction in primary metabolism observed in the current study could be part of the specific response to prolonged damage by insects. Similar results were reported in other studies (Gutsche et al., 2009; Donze-Reiner et al., 2017). Some authors consider that primary metabolism is reorganized to reallocate resources to defensive metabolic pathways whereas others consider the direct role of primary metabolites as defenses or signals (for review see Schwachtje and Baldwin, 2008). These two points of view could complement rather than contradict each other under the hypothesis of a changing maize response to insects over time, in which primary metabolites are crucial for developing primary defenses but lose priority as the damage is prolonged.

The second group of transcripts with the highest impact on the metabolome encompassed three transcripts. These transcripts were up-regulated after insect feeding and are involved in stress defenses and detoxification. Considering the whole transcriptomic and metabolomic data, we observed activation of the ROS scavenging machinery after *S. nonagrioides* feeding. This result is consistent with other studies in which up-regulation of ROS scavenging mechanisms was observed in the long-term defensive response to insect attack. Differences in ROS production were associated with differences in plant resistance (Gutsche et al., 2009). Related to the redox status of the plant, we identified seven transcripts with a cupin-like domain that were up-regulated and one that was repressed after attack by *S. nonagrioides.* These transcripts, with the exception of the one that was repressed, also showed the three characteristic protein domains of germin-like proteins (GLPs), including essential amino acids that form the *N*-glycosylation sites (Bernier and Berna, 2001). GLPs have three different activities: oxalate oxidase (OXO), superoxide oxidase (SOD), and protease inhibition (Ramputh et al., 2002). Ramputh et al. (2002) demonstrated the defensive role of GLPs against insect borers through transformation of a corn hybrid with a wheat GLP gene that reduced feeding by *O. nubilalis* in both *in vitro* and *in vivo* assays. Supporting the hypothesis of a defensive role against biotic stresses, the seven genes upregulated in our transcriptome analysis are localized in a cluster on chromosome 4 that shows synteny with a region on chromosome 8 of rice (*Oryza sativa* L.) comprising 12 highly conserved GLP genes involved in resistance against pathogen infection (Manosalva et al., 2009).

ROS production could be an effective defense against insect attack but detrimental effects of ROS on the plant itself should be prevented; detoxification and antioxidant compounds play significant roles in plant protection against ROS damage. We observed a significant decrease in the levels of reduced glutathione and L-Cys–Gly, which is an intermediate in the γ-glutamyl cycle of glutathione degradation, though this pathway seems to be marginal in plants (Ohkama-Ohtsu et al., 2008). The increased expression of several glutathione-*S*-transferases could explain the depletion of reduced glutathione since these enzymes are involved in the detoxification of xenobiotic compounds through their conjugation with glutathione. Our data also show that maize plants challenged with *S. nonagrioides* larvae accumulated higher amounts of DHA than control plants because DHA could not be back-reduced to AsA, probably due to the reduced amount of glutathione available. However, as AsA is essential for protecting the plant from ROS damage, a monodehydroascorbate (MDHA) reductase was over-expressed probably as a consequence of plant efforts to regulate the levels of AsA present in plant tissue.

### The Role of Insect Elicitors Is Marginal in the Long-Term Response

To discriminate the effect of the insect secretions on the activation of the long-term maize defenses, we treated maize plants with regurgitates obtained from caterpillars of *S. nonagrioides*. Despite the presence of well-characterized elicitors (such as Volicitin) in the insect regurgitates, the role of the caterpillar regurgitates in eliciting the late defensive response did not appear to be relevant. The transcriptomic responses to long-term feeding by *S. nonagrioides* and to caterpillar regurgitate challenge only shared changes in seven transcripts, though just one of them showed a similar pattern of response to both treatments, suggesting that the effect of elicitors present in the insect regurgitate is marginal compared to the effect of insect chewing. Consistent with this, the growth curves of larvae fed with stems from plants infested with *S. nonagrioides* differed significantly from those of larvae fed with plants treated with *S. nonagrioides* regurgitate upon wounding (unpublished data). However, a more relevant role of regurgitate in the early response of maize to stem feeding by borers cannot be ruled out due to some agreement between transcripts up-regulated in response to *S. nonagrioides* regurgitate and those reported previously as being implicated in the short-term response to feeding by *S. nonagrioides* (Rodríguez et al., 2012).

It is interesting to note that our metabolomics results support the hypothesis that some herbivores can avoid proper detection by actively depositing microbes in their secretions on plants (Felton et al., 2014). Plants challenged with *S. nonagrioides* regurgitate accumulated mycotoxins synthesized by fungus likely vectored by caterpillars and significantly reduced the amount of some metabolites that exhibit inhibitory activity against insect growth, such as viscutin 1 (Kubo et al., 1987). Therefore, part of the arsenal of transcripts and metabolites promoted in the plant by the insect regurgitate treatment could be oriented to defend against microbe attack rather than insect chewing.

## Conclusion

Different defensive mechanisms are involved in the long-term response to corn-borer feeding compared to those previously reported in the early response. The long-term response is characterized by reorganization of the primary metabolism and a strong redox response mainly mediated by GLPs to produce anti-nutritive and toxic compounds that reduce insect viability, the glutathione–ascorbate cycle being crucial to minimize the adverse effects of ROS. Few common features were observed in the late plant response to *Sesamia* and insect regurgitate challenges, suggesting that the role of insect elicitors is marginal in the long-term response.

## Author Contributions

VR and AB carried out the experiments, performed statistical analysis of the data, and drafted the initial manuscript. GP performed the bioinformatic analysis of the transcriptomic data. MK performed the metabolomics analysis. RM and RS assisted in the field experiment design, data collection, and statistical analysis. All authors read and approved the final version of the manuscript.

## Conflict of Interest Statement

The authors declare that the research was conducted in the absence of any commercial or financial relationships that could be construed as a potential conflict of interest.

## Footnotes

1 ftp://ftp.ensemblgenomes.org/pub/plants/release-23/fasta/zea_mays/

2 http://novocraft.com

3 http://kobas.cbi.pku.edu.cn/
