# Supplementary material for: Maize Stem Response to Long-Term Attack by Sesamia nonagrioides
Source: Front Plant Sci. 2018 Apr 23;9:522. doi: 10.3389/fpls.2018.00522 (PMC5925969; doi:10.3389/fpls.2018.00522)
Supplement: Supplementary file 3 [file Table_3.docx]

**Supplementary Table 3**. List of previously identified metabolites involved in the plant response to feeding of Lepidopteran larvae.

| METABOLITE | EXACT MASS |
| --- | --- |
| 2-Heptanonol ^1^ | 116.120 |
| α-Pinene ^1^ | 136.125 |
| Limonene ^1^ | 136.125 |
| α-Copaene ^1^ | 204.188 |
| Zingiberene ^1^ | 204.188 |
| α-Cedrene ^1^ | 204.188 |
| Cedrol ^1^ | 222.198 |
| JA ^1, 5^ | 210.126 |
| OPDA ^1^ | 292.204 |
| Me-JA ^1^ | 224.141 |
| SA ^1^ | 138.032 |
| IAA ^5^ | 175.063 |
| Ethylene ^5^ | 28.031 |
| DIMBOA-Glc ^2, 3, 4^ | 373.101 |
| HDMBOA-Glc ^2, 3, 4^ | 387.117 |
| Kauralexin A1 ^2^ | 303.466 |
| Kauralexin A2 ^2^ | 332.199 |
| Kauralexin A3 ^2^ | 317.212 |
| Kauralexin B1 ^2^ | 301.217 |
| Kauralexin B2 ^2^ | 330.183 |
| Kauralexin B3 ^2^ | 315.196 |
| Anthranilate ^5^ | 137.048 |
| Indole ^5^ | 117.058 |
| HDM_2_BOA-Glc ^2, 4^ | 417.128 |
| Coumaroyltyramine ^4^ | 283.121 |
| Tryptophan ^4^ | 204.087 |
| Feruloyltyramine ^4^ | 313.131 |
| HMBOA-Glc ^2, 4^ | 357.105 |
| DHBOA-Glc ^2, 4^ | 343.091 |
| DIBOA-Glc ^2, 4^ | 343.092 |
| DIMBOA ^2, 3, 4^ | 211.048 |
| DIM_2_BOA-Glc ^2, 4^ | 403.110 |
| TRIBOA-Glc ^2, 4^ | 359.086 |
| Maysin ^4^ | 576.149 |
| Rutin ^4^ | 610.153 |
| Kaempferol-rutinoside ^4^ | 594.159 |
| Galaptopyranosyl-linolenoylglycerol ^4^ | 514.315 |
| Digalaptopyranosyl-linolenoylglycerol ^4^ | 676.369 |
| Azelaic acid ^4^ | 188.105 |

^1^ Zhou et al. 2011. Physiologia Plantarum. 143: 21-40

^2^ Dafoe et al. 2013. PloS One. 8: e73394

^3^ Guo et al. 2017. Journal of Economic Entomology. 110: 739-747

^4^ Marti et al. 2013. Plant,Cell and Environment. 36: 621-639

^5^ Dafoe et al. 2011. Journal of Chemical Ecology. 37: 984-991
